# miR-758-3p/ILK signaling modulated angiogenesis by regulating VEGFA in wound healing

**DOI:** 10.7150/ijms.86733

**Published:** 2024-01-01

**Authors:** Rui Wang, Jing Jia, Lin Zhou, Xinxi Zhu, Zhishui Tang, Hailong Shen, Yifan Qiao, Gengrui Nan, Zhuangqun Yang, Wei Ma

**Affiliations:** 1 Department of Plastic, Cosmetic and Maxillofacial, The First Affiliated Hospital of Xi'an Jiaotong University, Xi'an, Shaanxi, China.; 2 Department of Medical Oncology, The First Affiliated Hospital of Xi'an Jiaotong University, Xi'an, Shaanxi, China.; 3 Xi'an Jiaotong University, Xi'an, Shaanxi, China.; 4 Department of Orthopedic, The First Affiliated Hospital of Xi'an Jiaotong University, Xi'an, Shaanxi, China.; 5 Department of stomatology, Jingbian county People's Hospital, Yulin, Shaanxi, China.

**Keywords:** wound healing, ILK, miR-758-3p, angiogenesis, fibroblast

## Abstract

Chronic wounds cause physical, psychological and economic damage to patients, while therapeutic choices are limited. ILK was reported to play key roles in both fibrosis and angiogenesis, which are two important factors during wound healing. However, the function of ILK during vascularization in wounds remains unclear. In our study, we found increased ILK expression in chronic wound tissues compared to adjacent tissue, as well as a positive relationship between ILK expression and microvessel density. Moreover, fibroblasts overexpressing ILK showed an enhanced ability to promote HUVEC migration and tube formation, during which PI3K/Akt, downstream of ILK, played key roles and VEGFA was the key cytokine. Considering the important function of ILK in wound healing and the lack of an ILK activator, we investigated microRNAs targeting ILK and found that miR-758-3p could target ILK to regulate its transcription. The inhibition of miR-758-3p increased ILK expression and sequentially upregulated VEGFA and activated angiogenesis *in vivo* and *in vitro*. Taken together, these results revealed that ILK played a key role in wound healing by regulating angiogenesis and that activating ILK by inhibiting miR-758-3p was an effective way to promote wound healing. Whether miR-758-3p/ILK signaling can be utilized as a therapeutic target for wound healing requires further investigation.

## Introduction

The integrity of the skin is very important for protecting the body from the external environment. The breakdown of integrity causes wounds and subsequent wound healing, while conditions including diabetes, malnutrition, and vascular occlusion cause chronic wounds. Chronic wounds cause psychological and physical damage and enormous costs to patients. However, very few treatment options have been presented for patients; thus, there is an urgent need for therapeutic approaches for chronic wounds^1^. Typically, fibroblasts play key roles in wound healing, while microcirculation also contributes greatly to the process^2^.

Angiogenesis is a process involving the formation of a novel capillary network to provide nutrients to cells^3^. Angiogenesis is regulated by a strict balance of different cytokines in the microenvironment. Cytokines secreted by fibroblasts, the main cellular component during wound healing, mediate endothelial cell activity and subsequently regulate angiogenesis^4^. Thus, the mechanism by which fibroblasts regulate the biological behavior of endothelial cells might affect wound healing.

ILK, an integrin-linked kinase, is located in focal adhesion complexes, which link to the cytoplasmic domain of integrin receptors to participate in the signal transduction of cells and the extracellular matrix. ILK involvement in fibrosis^5^ and vascular disorders^6^ has been verified. In addition, specifically ablating ILK in skin fibroblasts results in the defective formation of granulation tissue and delayed wound healing^7^. Deletion of ILK in cutaneous hair follicle stem cells leads to impaired skin repair^8^. Moreover, ILK increased angiogenesis to promote infarcted myocardium repair^9^. However, to our knowledge, the role of ILK in mediating angiogenesis during wound healing is unclear.

## Materials and Methods

### Chemicals and reagents

Primary antibodies against ILK (#3856), phospho-(p-)AKT (Ser473; #3787), AKT (#4691) and CD31 (#92841) were purchased from Cell Signaling Technology (Beverly, MA, USA). Primary antibody against VEGFA (Vascular Endothelial Growth Factor A, ab46154) was purchased from Abcam. QLT0267 was purchased from Sigma-Aldrich (St. Louis, MO, USA). X-treme GENE HP DNA transfection reagent was obtained from Roche (Mannheim, Germany). All reagents were stored and used according to the protocol provided by the manufacturer.

### Patients and Tissue Samples

Twenty-four chronic wound tissues and adjacent normal cutaneous tissues were collected from patients treated at the First Affiliated Hospital of Xi'an Jiaotong University. Tissues were embedded in paraffin prior to immunohistochemical (IHC) analysis of CD31, ILK and VEGFA expression. The protocol of tissue acquisition received approval from the Institutional Review Board. Informed consent was provided by each patient. Sections were derived from all tissue samples with a thickness of five microns prior to the corresponding preparation. The DAKO Autostainer Plus system was employed to analyze the expression of ILK (1:200), VEGFA (1:200) and CD31 (1:150).

The microvessel density was defined as the average number of microvessels from 5 random fields. Microvessels were defined as CD31^+^ endothelial cells that formed tubes or cell clusters detached from any microvessel structures. The staining scores of ILK and VEGFA were calculated by multiplying the intensity score by the percentage score. The intensity score was estimated as 0, 1, 2 and 3 for samples with no staining, weak positive staining, moderate positive staining and strong positive staining, respectively. The percentage score was estimated as 0, 1, 2, 3 and 4 for samples with positive percentages of 0%, ≤25%, 25-50%, 50-75% and ≥75%, respectively. In a double-blind manner, one pathologist quantified all sections under a high-power field (×400). The clinicopathological characteristics of the patients can be found in Table 1.

### Cell Culture and RNA Interference

Human skin fibroblasts were maintained in a humidified atmosphere with 5% CO_2_ at 37 °C and propagated in DMEM/F12 (Invitrogen; Thermo Fisher Scientific, Inc.) containing fetal bovine serum (Invitrogen; Thermo Fisher Scientific, Inc.). Reconstructed replication-defective lentiviruses harboring messenger ribonucleic acid (mRNA) fragments specifically overexpressing ILK or ILK control were transfected into fibroblasts; infected cells were identified as ILK-overexpressing (ILK-OV) and ILK-negative control (ILK-NC) cells. The efficiency of overexpression was detected by Western blot analysis. The miR-758-3p inhibitor and its negative control (NC) were purchased from RiboBio (Guangzhou, China) and were used to transfect cells together with X-treme GENE siRNA Transfection Reagent (Roche) when cells reached 30-50% confluence.

### Cell Counting Kit-8 (CCK8) assay

Cell Counting Kit-8 (CCK8, Dojindo Molecular Technologies, Inc.) was used to analyze cell viability and growth rate. Briefly, cells (4×10^3^) were seeded in 96-well culture plates for 24 hours. Then, the cells were differently treated for the indicated time periods, followed by a 3-hour incubation with serum-free medium (150 μl) containing 10% CCK8 at 37 °C. The medium was carefully discarded prior to dissolving the formazan crystals with 150 μl DMSO. Finally, we examined the absorbance with a microplate autoreader (Bio-Tek Instruments Inc.) at a wavelength of 450 nm. Independent experiments were repeated three times.

### Conditioned medium collection

Cells at 70-80% confluence were washed with serum-free medium three times before being cultured in serum-free medium for 24 hours. To obtain conditioned medium (CM), the cell culture supernatant was collected by centrifugation and filtration, followed by immediate use or storage until use at -80 °C.

### Wound healing assay

Fibroblasts were seeded into 6-well plates. After 24 h, a 10 μl sterile gun tip was used to vertically scratch the bottom of the well at the center, and the shed cells were washed with serum-free medium. Then, the cells were cultured with serum-free medium (SFM) or CM mixed with serum-free medium (1:1). Photos were taken at 0, 24 and 48 h to observe wound healing and calculate the rate of wound healing. Wound healing rate (%)=(0 h-scratch distance minus 24 h-scratch distance)/0 h-scratch distance × 100%.

### Transwell assay

A Transwell system with 8-μm-pore Transwell inserts (Millipore Corp., Billerica, MA, USA) was employed to analyze HUVEC (human umbilical vascular endothelial cell) migration. Briefly, 3×10^5^ cells were suspended in 300 μl of SFM, which was subsequently seeded into the upper chamber of the Transwell system. One milliliter of CM from differently treated fibroblasts was added to the lower chamber, followed by 16 h of culture. The cells that migrated to the lower surface of the inserts were fixed with 4% paraformaldehyde followed by staining with crystal violet. The visible cells were counted by one researcher in 5 random fields (200×) for each insert.

### Tube formation assay

A 24-well plate with Matrigel-coated wells was used for the tube formation assay. HUVECs (1×10^5^) were suspended in SFM or CM-supplemented SFM (1:1) prior to seeding into the Matrigel-coated wells. After culture for 6 h, imaging was taken using an optical microscope. The tube was identified as 2 perfectly connected branching points.

### Enzyme-linked immunosorbent assay (ELISA)

We employed the RayBio® Human VEGF ELISA kit (RayBiotech Inc., Norcross, GA, USA) to determine the concentration of A in CM collected from differently treated cells. Before detection, we modified the concentration of A by the addition of neutralizing A antibody or recombinant human VEGFA.

### RNA extraction and quantitative real-time PCR (RT‒qPCR)

The total RNA extraction kit purchased from Fastagen Biotech (Shanghai, China) was used to extract the total RNA before the subsequent reverse transcription using superscript III transcriptase (Invitrogen, Eugene, OR, USA). Then, the complementary DNA (cDNA) harvested from the reverse transcription was subjected to the RT‒qPCR assay using the Bio-Rad CFX96 system with SYBR Green. CT values were obtained using a qPCR platform (Bio-Rad CFX96 Real-Time PCR System). GAPDH was used for normalization. Relative gene expression was calculated by the 2^-ΔΔCt^ method. The primer sequences are shown in Supplementary Table I.

### Western blot assay

Protease inhibitor was added to the RIPA lysis buffer before lysing cells. The Bradford method was used to measure the protein concentration in the lysates. Protein samples (30 μg) were separated using electrophoresis in SDS-polyacrylamide gels prior to transfer to nitrocellulose membranes. Then, the membrane was incubated in 5% nonfat milk for 1 h at room temperature to block nonspecific binding sites. The blots were subjected to incubation with primary antibodies for 16 h at 4 °C, followed by incubation with secondary antibodies (anti-rabbit or anti-mouse antibody) for 1 h at room temperature. The ChemiDoc XRS molecular imager system (Bio-Rad Laboratories, Hercules, CA, USA) was used to photograph the protein bands with GAPDH as the loading control.

### Dual luciferase activity assay

The ILK promoter reporter plasmid pGL3-ILK was created by inserting 950 bp of its promoter, containing ILK-miR-758-3p binding sites, into the pGL3-basic plasmids. The mutant promoter of ILK was also designed and amplified, followed by insertion into the pGL3-basic plasmids, denoted pGL3-mutant (pGL3-mut). 293T cells and fibroblasts were transfected with pGL3-basic (pGL3-control), pGL3-mut or pGL3-ILK using DNA transfection reagent. After transfection with miR-758-3p, cells were subjected to a dual luciferase assay using a dual luciferase assay kit (Promega, Madison, WI, USA) following the instructions of the manufacturer. The average value from six independent wells was used for analysis. Independent experiments were repeated three times.

### The miR-758-3p inhibitor activated ILK to promote wound healing in vivo

A total of 24 8-week-old mice, half male and half female, were randomly allocated to 4 groups with random digits. The mice were fed with free access to water and food at room temperature (22+/-1℃) with a humidity of 40+/-10% and a light cycle of 12/12 h. Under aseptic conditions, the mice were treated with 10% chloral hydrate (3.0 ml/kg), followed by the creation of a scald with a constant temperature and pressure apparatus. A deep II° wound surface was constructed by being burned for 8 seconds with a cylindrical test tube with a base area of 1×1 cm^2^ and a weight of 0.5 kg at 80 °C. Mice in different groups were treated with miR-758-3p inhibitor, miR-758-3p NC, QLT0267 (0.1 mg/kg) or DMSO. The miR-758-3p inhibitor and inhibitor-NC (20 µl) as well as DMSO and QLT0267 were injected subcutaneously at the wound edge for 2 consecutive days. We measured the wound and took microtissue from the wound on the 3rd, 7th, 14th and 21st days and harvested the whole skin of the back wound with suturing of the wound sequentially under anesthesia. The microtissues were preserved in liquid nitrogen before RNA extraction and protein extraction. The whole wound tissues were fixed with 4% paraformaldehyde, embedded in paraffin, and subjected to H&E staining and IHC staining of CD31 and ILK expression. The animal experiments were approved by the institutional review board of the First Affiliated Hospital of Xi'an Jiaotong University.

### Statistical analysis

GraphPad Prism (version 5.0) software was employed for the statistical analyses. Student's t test was used for two-group comparisons, and one-way ANOVA and Fisher's least significant difference t test (LSD t test) were used to compare ≥3 groups. For correlation analysis, Spearman's correlation test was introduced with SPSS (SPSS for Windows 10.0, SPSS, Inc.). P<0.05 was considered to indicate a statistically significant difference.

## Results

### The expression of ILK was increased and positively related to vascularization in chronic wound tissue

**Figure 1:**
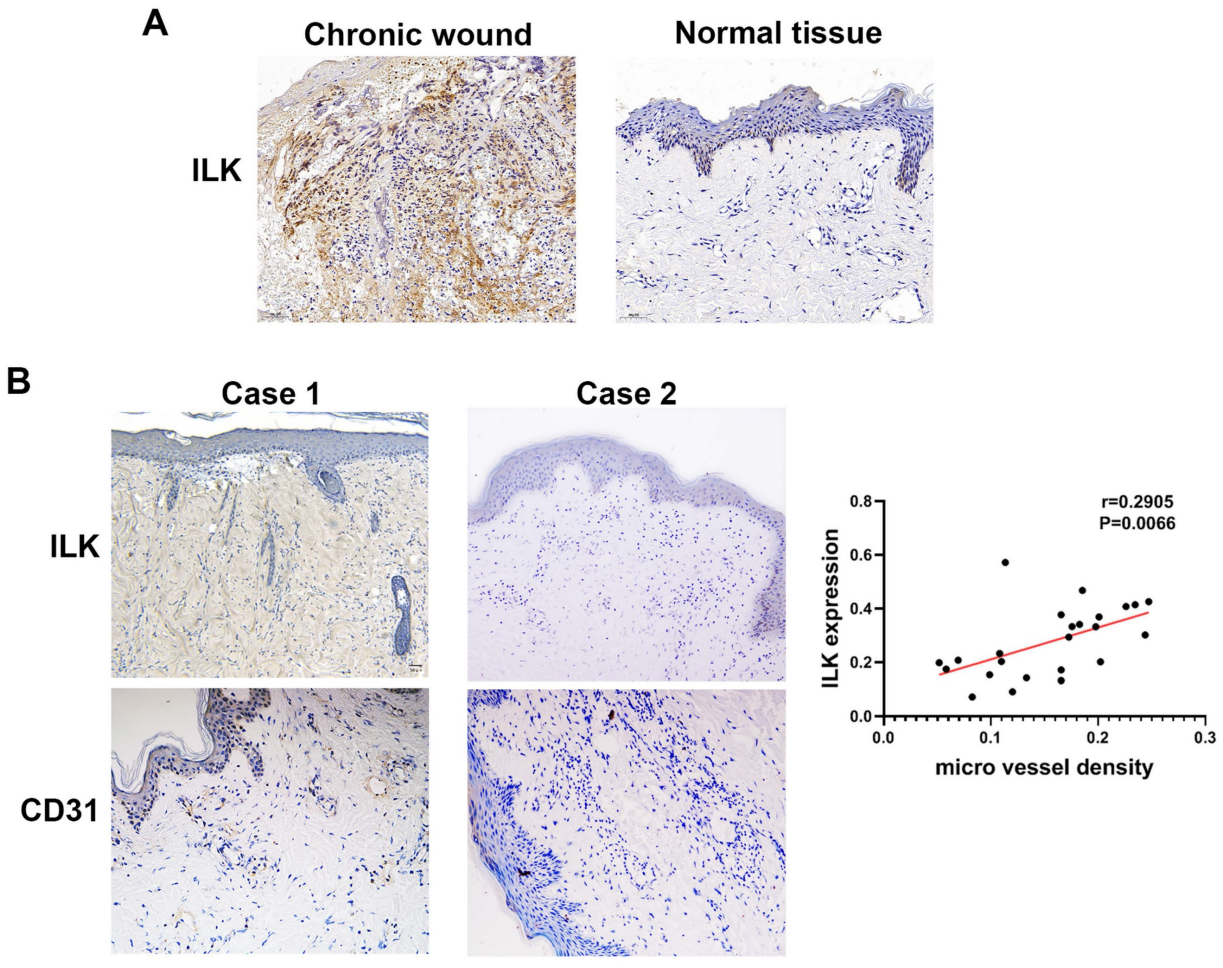
** Increased ILK in chronic wound tissue was positively related to MVD.** Immunohistochemical analysis was performed to analyze the expression of CD31 and ILK in chronic wound tissues. All sections were analyzed in a double-blind manner under a high-power field (×400). One pathologist counted the number of CD31^+^ cell clusters in each section, and the average number of vessels counted in 10 random fields was defined as the microvessel density (MVD). **A.** Representative images of ILK expression in chronic wound tissues and adjacent normal skin. **B.** Representative images of chronic wound samples with high ILK expression-high MVD and low ILK expression-low MVD. **C.** Correlation of MVD and ILK expression analyzed by linear regression analysis. *P<0.05.

To elucidate the function of ILK in promoting angiogenesis and wound healing, we performed IHC assays in 25 chronic wound tissues and adjacent normal tissues with antibodies against CD31 (a molecular marker of vascular endothelial cells) and ILK. The results of IHC staining showed increased ILK expression in chronic wound tissues compared with adjacent normal tissue (Figure 1A). Furthermore, chronic wound tissue with a high level of ILK expression also showed increased microvessel density. Further analysis revealed a positive correlation between ILK expression and microvessel density (r=0.6859, P<0.001, Figure 1B and 1C). The results above indicated that ILK was upregulated in chronic wound tissues, which potentially promoted angiogenesis during wound healing.

### Fibroblasts with upregulated ILK promoted angiogenesis

**Figure 2:**
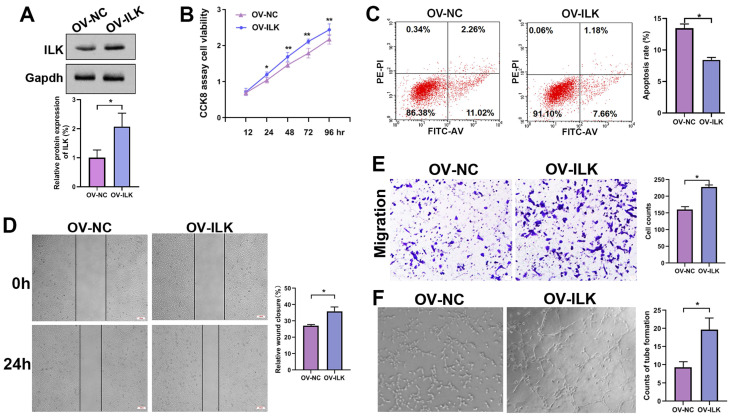
** ILK-overexpressing fibroblasts enhanced angiogenesis. A.** Protein level of ILK in fibroblasts with or without transfection of lentivirus overexpressing ILK; Lower panel: quantitative results of Western blot assay. **B.** CCK8 assay of HUVEC viability after treatment with CM from ILK-OV fibroblasts or negative control fibroblasts. **C.** Apoptosis detection of the aforementioned cell clones. **D.** HUVECs treated with CM collected from ILK-OV fibroblasts or iILK-NC fibroblasts were subjected to a wound healing assay to test the migration of HUVECs. **E.** HUVECs were seeded in the upper chamber with CM from ILK-OV fibroblasts or ILK-NC fibroblasts added to the lower chamber in the Transwell system. HUVECs that migrated to the lower surface of the upper chamber were counted in 5 random fields and analyzed. Right panel: quantitative results of recruited HUVECs. **F.** HUVECs were diluted using serum-free medium (SFM) with CM from ILK-OV fibroblasts or ILK-NC fibroblasts before seeding into Matrigel-coated wells. After incubation for 4 h, the tube number in each well was counted. The tube was identified as 2 branches perfectly connected. Left: representative images of tubes; right: quantitative data of tube number. *p<0.05.

For further study, we constructed ILK-OV and its negative control (NC) fibroblasts using lentivirus-delivered specific mRNA (Figure 2A). Then, we collected conditioned medium (CM) from ILK-OV and ILK-NC fibroblasts to treat HUVECs. The CCK8 assay showed increased cell viability in HUVECs treated with CM from ILK-OV fibroblasts compared to HUVECs treated with CM from ILK-NC fibroblasts (Figure 2B). Additionally, fewer apoptotic cells were found in ILK-OV-treated HUVECs, as demonstrated by the flow cytometry assay (Figure 2C). Moreover, a wound healing assay showed accelerated HUVEC migration after treatment with CM from ILK-OV fibroblasts (Figure 2D). Consistently, Transwell analysis further confirmed the enhanced migration of HUVECs induced by ILK-OV fibroblasts with CM from ILK-OV fibroblasts in the lower chamber (Figure 2E). In addition, a higher capability for tube formation was found in HUVECs treated with CM from ILK-OV fibroblasts (Figure 2F). Overall, our results demonstrated that fibroblasts with upregulated ILK showed a stronger ability to interact with HUVECs and subsequently promoted angiogenesis.

### ILK increased angiogenesis by regulating VEGFA in fibroblasts

**Figure 3:**
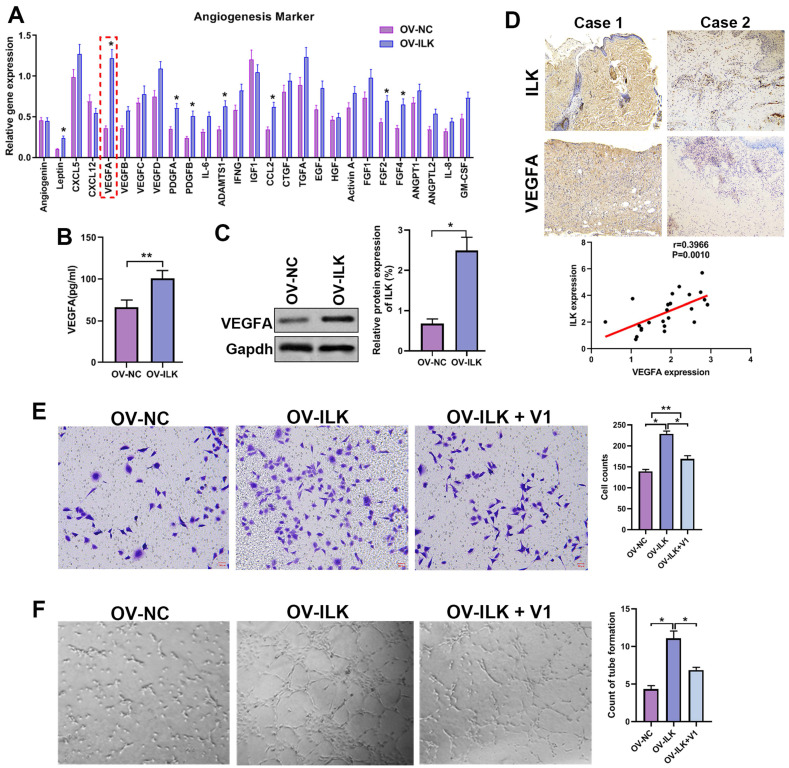
** A played a crucial role in ILK-mediated angiogenesis. A.** Expression of 40 proangiogenic cytokines detected by qPCR assay in ILK-OV and ILK-NC fibroblasts. **B.** CM collected from ILK-OV and ILK-NC fibroblasts was subjected to ELISA to explore the secretion of VEGFA. **C.** Protein level of VEGFA in fibroblasts with or without ILK overexpression; Right panel: quantitative results of Western blot assay. **D.** Representative images of samples with high ILK expression-high VEGFA expression and low ILK expression-low VEGFA expression. Correlation of VEGFA expression and ILK expression. **E.** CMs were collected from ILK-NC fibroblasts and ILK-OV fibroblasts with or without treatment with a VEGFA inhibitor (V1, 0.3 μM, 24 h). CM from differently treated fibroblasts was added to the lower chamber of a Transwell system with HUVECs seeded in the upper chamber. Left: representative images of recruited HUVECs; right: quantitative data of recruited HUVECs. **F.** HUVECs were diluted with SFM and treated with CM from fibroblasts at the head of the seeding into Matrigel-coated wells for tube formation assays. Right: quantitative data of the formed tube. *p<0.05, **p<0.001.

Since ILK-OV fibroblasts promoted angiogenesis by interacting with HUVECs, we hypothesized that ILK facilitated the expression and secretion of proangiogenic factors to implement this effect. Thus, we detected the expression of 40 proangiogenic cytokines, which indicated that VEGFA might be a key player participating in angiogenesis of ILK-OV fibroblasts (Figure 3A). To identify the upregulation of VEGFA with ILK overexpression, we employed ELISA and Western blot assays and found facilitated expression and secretion of VEGFA in ILK-OV fibroblasts (Figure 3B and 3C). To further confirm this result, we analyzed the expression of VEGFA in chronic wound tissues and found a high density of VEGFA in samples with higher levels of ILK expression. Statistical analysis revealed that VEGFA was positively correlated with ILK expression and microvessel density (Figure 3D). To verify the key role of VEGFA in ILK-mediated regulation of angiogenesis, we used V1, an inhibitor of VEGFA, to treat ILK-OV fibroblasts and collected CM from these cells. The results from the transwell assay revealed that inhibiting VEGFA weakened HUVEC migration induced by ILK overexpression (Figure 3E). Consistently, CM from ILK-OV fibroblasts enhanced tube formation of HUVECs, while suppressing VEGFA in ILK-OV fibroblasts reversed this enhancement (Figure 3F). Together, our results suggest that VEGFA plays a key role in ILK-facilitated angiogenesis.

### The mechanism underlying ILK-enhanced angiogenesis was active PI3K/Akt signaling

**Figure 4:**
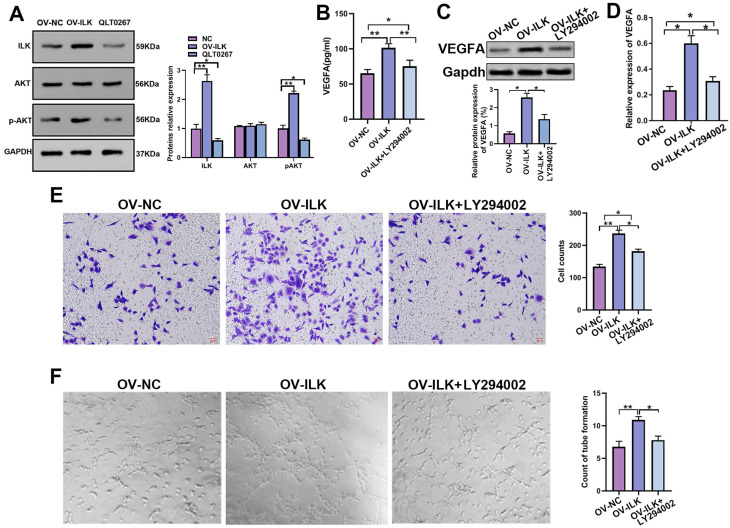
** PI3K/Akt signaling is the major mechanism by which ILK regulates wound healing. A.** Expression of p-Akt in ILK-NC-, ILK-OV- and QLT0267-treated fibroblasts; Right panel: quantitative results of Western blot assays. Fibroblasts were treated with QLT0267 (5 μM) for 24 h. **B.** ELISA, **C.** Western blot assay (lower panel: quantitative results of Western blot assay) and **D.** qPCR assay tested the expression of VEGFA in ILK-NC fibroblasts and ILK-OV fibroblasts with and without LY294002 treatment (10 μM, 24 h). **E.** CMs were collected from ILK-NC fibroblasts and ILK-OV fibroblasts with and without LY294002 treatment (10 μM, 24 h). CM from differently treated fibroblasts was added to the lower chamber of a Transwell system with HUVECs seeded in the upper chamber. Left: representative images of recruited HUVECs; right: quantitative data of recruited HUVECs. **F.** HUVECs were diluted with SFM and treated with CM from differently treated fibroblasts ahead of seeding in Matrigel-coated wells for tube formation assays. Right: quantitative data of the formed tube. *p<0.05, **p<0.001.

ILK is a kinase linked to various kinds of signaling pathways to implement its functions, and PI3K/Akt signaling is widely thought to be a crucial part of ILK downstream signaling in wound healing. We performed a Western blot assay that showed upregulated p-Akt in ILK-OV fibroblasts and downregulated p-Akt after treatment with QLT0267, an inhibitor of ILK, suggesting that ILK activated PI3K/Akt signaling in fibroblasts (Figure 4A). Next, we employed LY 294002 (an inhibitor of PI3K/Akt signaling) to treat ILK-OV cells. The results of the qPCR assay, Western blot assay and ELISA suggested that inhibiting PI3K/Akt signaling attenuated the increase in VEGFA expression and secretion induced by ILK overexpression (Figure 4B, 4C and 4D). Uniformly, CM from ILK-OV fibroblasts with PI3K/Akt inhibition displayed weak promotion of HUVEC migration compared with CM from ILK-OV fibroblasts without PI3K/Akt inhibition (Figure 4E). In addition, a tube formation assay proved that suppressing PI3K/Akt signaling impaired the ability of ILK-OV fibroblasts to promote tube formation (Figure 4G).

### Inhibiting miR-785-3p was an efficient way to activate ILK

**Figure 5:**
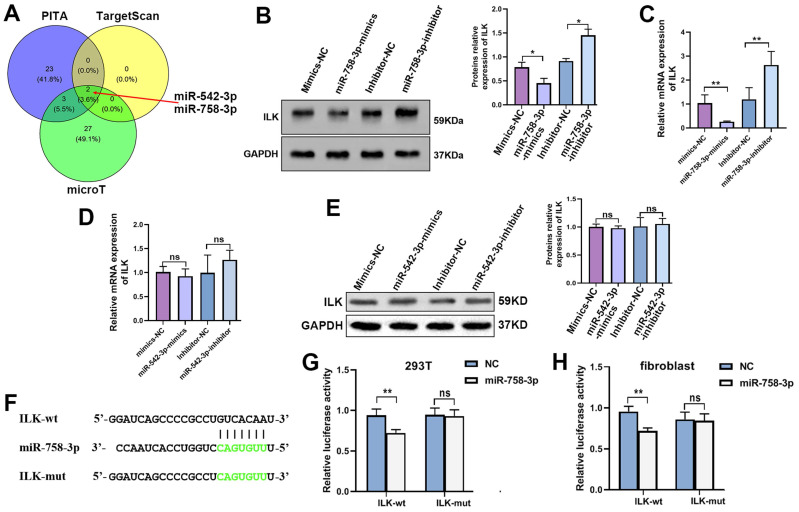
** MiR-758-3p could target ILK to regulate its expression. A.** Venn diagram of predicted microRNAs potentially targeting ILK with data from PITA, targetScan and microT. **B.** Protein level of ILK in fibroblasts treated with the miR-758-3p inhibitor or miR-758-3p mimics; Right panel: quantitative results of Western blot assay. **C.** Expression of ILK using qPCR assay in fibroblasts treated with the miR-758-3p inhibitor or miR-758-3p mimics. **D** and **E.** Protein and mRNA levels of ILK in fibroblasts treated with miR-542-3p inhibitor or miR-542-3p mimics. Lower panel: quantitative results of Western blot assay. **F.** Schematic diagram of the nuclear sequence in the 3' untranslated area of the ILK promoter targeted by miR-758-3p. **G.** pGL3-control, pGL3-ILK (harboring the fragment of the ILK promoter) or pGL3-mut (harboring the mutant ILK promoter) plasmids together with miR-758-3p mimics were transfected into 293T cells prior to the dual luciferase activity assay. **H.** The above plasmids and miR-758-3p were transfected into fibroblasts before the initiation of the dual luciferase activity assay. **p<0.001, ns: not significant.

Due to the important roles of upregulated ILK in various kinds of diseases, especially cancer, inhibitors of ILK have been discovered and used extensively^10^. However, few activators of ILK are currently available. Regarding the function of ILK overexpression in accelerating wound healing, we tried to find a way to upregulate ILK expression. MicroRNAs downregulate gene expression by specifically binding to their target genes. Thus, we predicted microRNAs potentially targeting ILK using the online database ENCORI to download the targeting-ILK miRNA data. The common miRNAs targeting ILK in the PITA, TargetScan and microT databases analyzed by the R package were miR-542-3p and miR-758-3p (Figure 5A). Next, we transfected inhibitors of miR-542-3p and miR-758-3p into fibroblasts and found that inhibiting miR-758-3p successfully upregulated ILK expression at both the mRNA and protein levels (Figure 5B and 5C). However, the miR-542-3p inhibitor failed to affect ILK expression in fibroblasts (Figure 5D and 5E).

Hence, we projected the miR-758-3p-targeting nuclear sequence in the 3' untranslated area of the ILK promoter in the starBase database (Figure 5F), which was then cloned and inserted into the pGL3-basic luciferase reporter plasmid to create the pGL3-ILK plasmid. In the same way, we designed a mutant sequence of the miR-758-3p-targeting nuclear sequence and constructed the pGL3-mut plasmid. The dual luciferase assay in 293T cells and fibroblasts indicated that miR-758-3p significantly enhanced the activity of pGL3-ILK without affecting the activity of pGL3-mut (Figure. 5G and 5H), which confirmed the binding of miR-758-3p and ILK. The results above suggested that miR-758-3p could target ILK to mediate its expression and that inhibiting miR-758-3p was an efficient way to overexpress ILK.

### MiR-758-3p upregulates ILK and promotes angiogenesis

**Figure 6:**
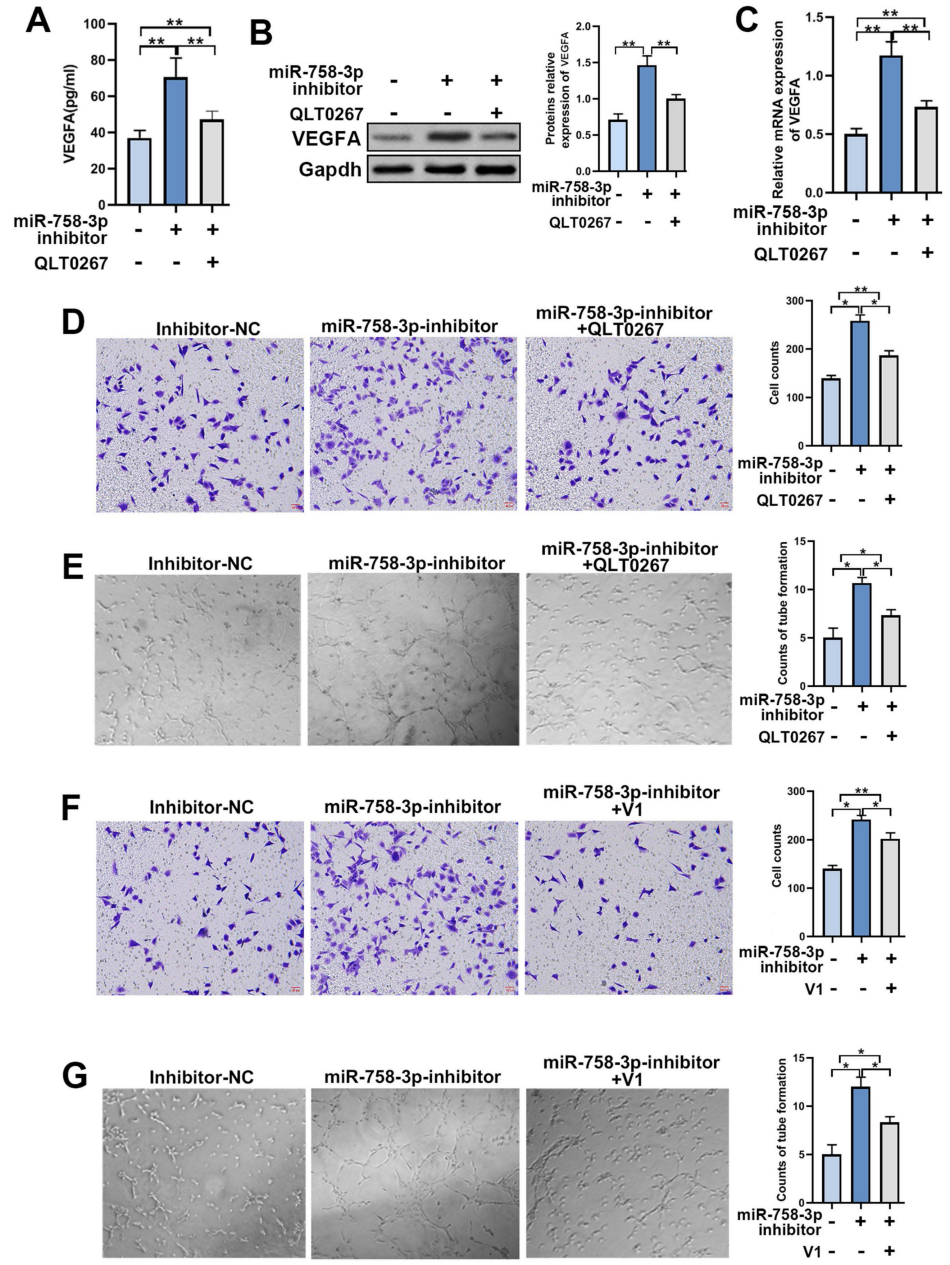
** The miR-758-3p inhibitor increased ILK and its function of promoting angiogenesis.** We treated fibroblasts with miR-758-3p inhibitor or miR-758-3p control for 24 h prior to treating miR-758-3p-inhibited cells with QLT0267 (5 μM) for another 24 h. Then, the expression of VEGFA was analyzed by **A.** ELISA, **B.** Western blot assay (right panel: quantitative results of Western blot assay) and **C.** qPCR assay. **D.** CMs were collected from miR-758-3p inhibitor-treated fibroblasts with or without QLT0267 treatment (5 μM, 24 h) and miR-758-3p control-treated fibroblasts. HUVECs treated with CM from differently treated fibroblasts were subjected to Transwell assays to test HUVEC migration; Right: quantitative data of migrated HUVECs. **E.** HUVECs diluted with SFM accompanied by CMs from differently treated fibroblasts prior to seeding into the Matrigel-coated well for the tube formation assay; Right: quantitative data of tube formation. **F.** CMs were collected from miR-758-3p inhibitor-treated fibroblasts with or without V1 treatment (0.3 μM, 24 h) and miR-758-3p control-treated fibroblasts. Then, CMs were added into the lower chamber of the Transwell system to detect the migration of HUVECs; Right: quantitative data of migrated HUVECs. **G.** HUVECs diluted with SFM accompanied by the aforementioned CMs prior to seeding into the Matrigel-coated well for the tube formation assay; Right: quantitative data of tube formation. *p<0.05, **p<0.001.

To further confirm the efficiency of miR-758-3p inhibition in upregulating ILK, we examined the expression and secretion of VEGFA in fibroblasts treated with the miR-758-3p inhibitor. The results from the qPCR assay, Western blot assay and ELISA showed that miR-758-3p inhibition increased VEGFA expression, while suppressing ILK via its inhibitor, QLT0267, relieved the enhancement of VEGFA expression (Figure 6A, 6B and 6C).

To test the functionality of the miR-758-3p inhibitor upregulating ILK, we treated HUVECs with CM from miR-758-3p-inhibiting fibroblasts with or without treatment with QLT0267 and performed a Transwell assay. The results proved that inhibiting miR-758-3p accelerated HUVEC migration, while suppressing ILK reversed that acceleration (Figure 6D). Additionally, HUVECs treated with CM from fibroblasts with miR-758-3p inhibition formed more tubes, and ILK suppression destroyed the facilitated tube formation ability caused by the miR-758-3p inhibitor (Figure 6E). Consistently, inhibition of VEGFA by adding its inhibitor, V1, successfully tempered miR-758-3p inhibitor-induced HUVEC migration and tube formation (Figure 6F and 6G). These results demonstrated that the miR-758-3p inhibitor promoted angiogenesis by increasing VEGFA.

Taken together, the results from Figure 6 suggested that miR-758-3p could successfully upregulate ILK and further work as an ILK activator to promote angiogenesis.

### A miR-758-3p inhibitor, as an ILK activator, accelerated wound healing, and suppressing ILK delayed wound healing in mice

**Figure 7:**
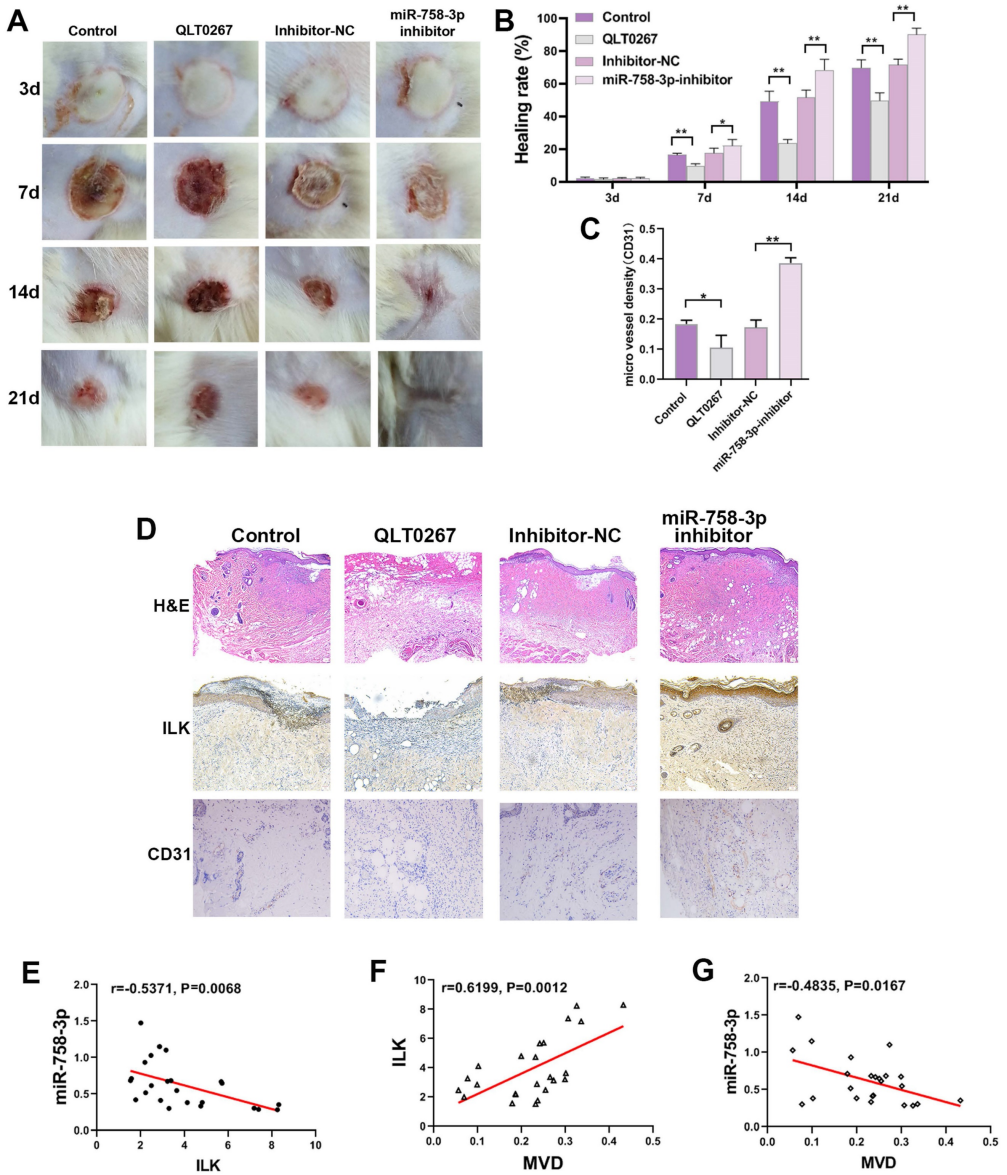
** The miR-758-3p inhibitor, as an effective ILK activator, accelerated wound healing in mice. A.** The general appearance of the scald wounds with different treatments on the 21^st^ day. **B.** The healing rate of the differently treated scald wounds on the 21^st^ day. The healing rate was identified as the percentage of contraction ((the initial area minus the initial area)/the initial area). **C.** Representative images of IHC assays with antibodies against ILK and CD31 (molecular markers of vascular endothelial cells) of wound samples in different groups. **D.** Quantitative results of MVD in different groups. **E, F** and **G.** The relationships of miR-758-3p and ILK expression, ILK expression and MVD, and miR-758-3p expression and MVD were analyzed by Pearson correlation analysis in all mice on the 21^st^ day.

To further confirm the efficiency of miR-758-3p in promoting wound healing, we created a deep II° wound surface on the depilated back of 24 mice. Then, the mice were randomly divided into four groups and treated with miR-758-3p inhibitor, inhibitor-NC, DMSO and QLT0267 (0.1 mg/kg). Beginning on the 7^th^ day, the wound healing rate was significantly higher in the group treated with the miR-758-3p inhibitor, while a lower wound healing rate was found in the group treated with QLT0267 (Figure 7A and 7B). At the micro level, compared to the DMSO group, severely porous and vacuolated fibrous tissue accompanied by inflammatory cell-infiltrated epidermis was observed in the group treated with QLT0267. In contrast, compared to the inhibitor-NC group, the wound site was well recovered with obvious hyperplastic dermis in the miR-758-3p-inhibitor group (Figure 7C). Similarly, increased microvessel density was found in the miR-758-3p-treated group, while few microvessels were found in the group treated with QLT0267 (Figure 7C and 7D). Furthermore, we examined the expression of miR-758-3p and ILK as well as microvessel density in the microtissues taken from different groups on the 21^st^ day. Pearson correlation analysis showed a negative correlation between miR-758-3p and ILK (r = -0.5371, p=0.0068, Figure 7E). Moreover, a positive correlation was found between ILK expression and microvessel density (r =0.6199, p=0.0012, Figure 7F), and a negative correlation was found between miR-758-3p and microvessel density (r = 0.4835, p=0.0167, Figure 7G).

The supervision of miR-758-3p inhibitor efficiency was performed by taking microtissues from different time points in the miR-758-3p-inhibitor group, which indicated dynamic changes in early increases and late decreases in ILK expression and an opposite trend in miR-758-3p expression (SFigure 1). The reason for the damaged positive correlation between ILK expression and microvessel density on the 21^st^ day might be that the expression of ILK decreased with loss-of-function of the miR-758-3p inhibitor; however, the formed vessels did not disappear.

The results in mice revealed the important role of ILK in wound healing and the role of the miR-758-3p inhibitor in promoting wound healing, which might be a potential treatment option for wound therapy.

## Discussion

In this study, our results showed that CM from ILK-overexpressing fibroblasts could promote upregulated cell viability, migration and tube formation in HUVECs and hence promoted angiogenesis to accelerate wound healing. The underlying mechanism of ILK overexpression in fibroblasts enhancing angiogenesis was that ILK increased VEGFA expression and secretion by activating PI3K/Akt signaling. Moreover, we found that inhibiting miR-758-3p, which targets ILK to decrease its expression, could upregulate ILK and subsequently enhance wound healing. Therefore, an inhibitor of miR-758-3p could be a potential therapeutic treatment option for patients with chronic wounds.

ILK plays an essential role in conserved intracellular signaling by forming the ILK-PINCH-Parvin (IPP) complex. Through binding to integrins and the IPP complex, ILK is associated with various signaling pathways, including cell adhesion, migration, proliferation and other common cellular functions. ILK implements these functions by mediating a plethora of signaling pathways, among which PI3K/Akt signaling and its downstream effectors mTOR and GSK3-β are the most widely studied^11^. A study using ILK knockdown mice verified that a lack of ILK delayed wound healing, during which ILK/PI3K/Akt signaling played an important role^12^. In addition, ILK activated NF-κB and upregulated the transcription of NF-κB-dependent genes to promote angiogenesis^13^. Overexpression of ILK in HUVECs accelerated cell proliferation, migration and tube formation by regulating TGFβ and SMAD2^14^. Moreover, Shafiei et al. proved that ILK enhanced cell migration and adhesion to promote the restoration of injured liver^15^. In our study, we demonstrated that increased ILK activated downstream PI3K/Akt signaling in fibroblasts, which recruited HUVECs and promoted proliferation and tube formation. Well-created neovascularization provided a good safeguard for wound healing. With constant discovery of the important functions of ILK, inhibitors of ILK were discovered. However, few activators of ILK could be used for therapy.

Mass spectrometry data with high-throughput analysis revealed that dozens of posttranslational modifications influenced the function of ILK, including phosphorylation, acetylation, ubiquitination, sumoylation, and methylation. The export of ILK from the nucleus into the cytoplasm is regulated by PAK1 by mediating ILK phosphorylation^16^. The TATA-less and GC-rich area in the promoter of ILK allows transcription factors, such as AP-2 and Sp1, to bind and manage ILK expression^16^, ^17^. In addition, a growing body of evidence supports the finding that microRNAs can bind to the 3'-terminus of ILK to inhibit its expression. miR-542-3p, miR-625 and miR-145 binding to IKL and sequentially affecting its function have been verified^18^-^20^. In this study, we demonstrated that miR-758-3p targeted ILK to diminish its expression and that suppressing miR-758-3p was an effective way to increase ILK expression and its role in enhancing vascularization during wound healing.

MicroRNAs (miRNAs) are evolutionarily conserved noncoding RNAs containing 20-24 nucleotides^21^. Many studies have proven that miRNAs are potential biomarkers for prediction, diagnosis and even targets of therapy in various kinds of diseases, including chronic wound healing^22^, ^23^. For example, targeting miR-133b restored EGFR to promote wound healing by upregulating angiogenesis^24^. MiR-758-3p has been shown to play an important role in regulating cell proliferation, migration and invasion in many kinds of tumors^25^, ^26^. Moreover, miR-758-3p has been found to inhibit cell viability and tube formation in aortic endothelial cells^27^. Considering the therapeutic value of miR-758-3p and its function of targeting ILK, we used miR-758-3p to treat mice with wounds. The results of our study showed that inhibiting miR-758-3p significantly accelerated wound healing and upregulated ILK expression, which indicated that miR-758-3p might be used as an ILK activator to promote wound healing.

In conclusion, we found that overexpressing ILK in fibroblasts promoted angiogenesis to accelerate wound healing. In addition, we also demonstrated that inhibiting miR-758-3p was an effective way to activate ILK, which might be a potential treatment option for patients with chronic wounds.

## Supplementary Material

Supplementary figure and table.

## Figures and Tables

**Table 1:** Clinicopathologic characteristics of patients

Characteristic	Number (%)
Total	24
Age	
≤40 years	8 (33.3%)
>40 years	16 (66.7%)
Gender	
Male	13 (54.2%)
Female	11 (45.8%)
BMI; kg/m^2^	
<18.5	3 (12.5%)
≥18.5, <23.9	7 (29.2%)
≥23.9, <27.9	8 (33.3%)
≥27.9	6 (25%)
Wound duration, weeks	
<12	5 (20.8%)
≥12, <24	7 (29.2%)
≥24	12 (50%)
HbA1c, %	
≤6.5	10 (41.7%)
>6.5	14 (58.3%)
ALB, g/L	
<35	15 (62.5%)
≥35	9 (37.5%)

## Abbreviations

ILK: integrin-linked kinase
cRNA: complementary ribonucleic acid
siRNA: small interfering ribonucleic acid
mRNA: messenger ribonucleic acid
VEGFA: vascular endothelial growth Factor A
CCK8: Cell Counting Kit-8
CM: conditioned medium
SFM: serum-free medium
luci: luciferase assay
cDNA: complementary DNA
IHC assay: immunohistochemical assay
qRT‒PCR: real-time quantitative-polymerase chain reaction
OV: overexpressed
NC: negative control
ELISA: enzyme-linked immunosorbent assay
HUVEC: human umbilical vein endothelial cells
mut: mutant
