# Supplementary material for: miR-758-3p/ILK signaling modulated angiogenesis by regulating VEGFA in wound healing
Source: Int J Med Sci. 2024 Jan 1;21(1):175–87. doi: 10.7150/ijms.86733 (PMC10750343; doi:10.7150/ijms.86733)
Supplement: Supplementary file 1 — Supplementary figure and table. [file ijmsv21p0175s1.pdf]

Supplementary Material

Supplementary Figures

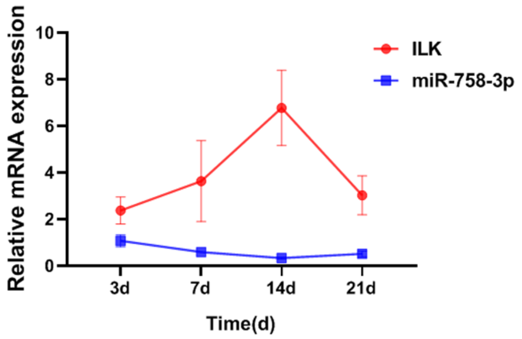

Supplementary Figure 1. Expression of miR-758-3p and ILK in miR-758-3p treating group in different times during wound healing.

Supplementary Table I.

Gene		Primer (5’-3’)
CD31	F	GAAAGACAAGGCGATCGTGG
	R	CAGGTCCAGCATTTCCCCTT
CD33	F	GAGGAGGCCTTGTGTGTTCA
	R	GTGCATCTCTGATGAGCAGG
ILK	F	AAGGTGCTGAAGGTTCGAGA
	R	TGGCATCCAGTGTGTGATGA
VEGFA	F	TGTGCGCAGACAGTGCTC
	R	TTCTGCTCCCCTTCTGTCGT
GAPDH	F	GCGAGATCCCGCTAACATCA
	R	TCACAAACATGGGGGCATCA
miR-758-3p	F	TTTGTGACCTGGTCCACTAACC
	R	GTGCGTGTCGTGGAGTCG
